# Windows-APT 2025: A dataset for APT-inspired attack scenarios on windows systems

**DOI:** 10.1016/j.dib.2026.112569

**Published:** 2026-02-11

**Authors:** Maryam Mozaffari, Abbas Yazdinejad, Ali Dehghantanha

**Affiliations:** a Cyber Science Lab (CSL), Guelph, ON, Canada; b AIMMLab, Dalla Lana School of Public Health, University of Toronto, Toronto, ON, Canada; c DCAILab, Department of Computer Science, University of Regina, Regina, SK, Canada

**Keywords:** Advanced Persistent Threat, MITRE ATT&CK, Intrusion Detection, Tactics Techniques and Procedures, Cybersecurity Dataset

## Abstract

The Windows-APT Dataset 2025 represents a significant advancement in cybersecurity research, addressing critical gaps in the understanding of advanced persistent threat (APT) tactics against Windows systems. Existing datasets largely focus on network data, often overlooking the detailed tactics, techniques, and procedures (TTPs) used by sophisticated threat actors. To bridge this gap, we developed a comprehensive dataset of 36 APT-inspired scenarios derived from threat actor profiles documented in the MITRE ATT&CK framework. Scenario selection mirrors MITRE ATT&CK group entries reported as China-attributed; we do not assert attribution and focus strictly on reproducing reported TTPs for research. Leveraging the MITRE Caldera framework for adversary emulation, we generated extensive system and network event logs, collected via Wazuh, and systematically mapped them to the MITRE ATT&CK framework. This dataset provides a valuable asset for machine learning model training, intrusion detection system evaluation, and the enhancement of APT dynamics studies. By providing a detailed view of APT activities in Windows environments, it enables stronger threat detection, informs defensive strategies, and facilitates development of effective countermeasures against emerging cyber threats. The dataset package contains 19 CSV files (including 16 per-period logs, one combined log, and two supplementary CSVs for manifest and validation), along with configuration files to support exact replication.

Specifications Table

**Table:** 

Subject	Computer Science/Cryptography and Cybersecurity
Specific subject area	APT-inspired attacks in Windows environments, derived from MITRE ATT&CK group profiles; no attribution is asserted.
Type of data	Comma-separated values (CSV)
Data collection	A Windows environment was created to simulate 36 scenarios inspired by threat actor profiles in the MITRE ATT&CK framework. Many profiles are discussed in open-source intelligence; we replicate only reported TTPs. Before executing scenarios, security solutions were installed and configurations were validated. The dataset captures the TTPs used by these profiles, enabling a detailed look at attacker behaviors.
Data source location	Data were collected on a centralized security system known as SIEM (Wazuh), configured to gather detailed logs and confirm all necessary integrations before performing simulations.
Data accessibility	Repository name: Mendeley Data DOI: 10.17632/b8fmtzvpy8.3 [1] Direct URL to data: https://data.mendeley.com/datasets/b8fmtzvpy8/3 License: CC BY 4.0 Integrity: SHA-256 checksums for all CSVs are provided in checksums.sha256. Package contents: 19 CSV files (16 per-period log CSVs, 1 combined log CSV, scenario_manifest.csv, validation_summary.csv).
Related research article	None

## 1 Value of the Data

The Windows-APT Dataset 2025 is a pivotal resource for advancing research on APTs against Windows environments. It addresses a long-standing gap in publicly available datasets, which tend to focus on network traffic and omit the complete range of TTPs observed in real-world APT campaigns. Based on threat actor profiles reported in the MITRE ATT&CK framework, some publicly reported in open-source intelligence as attributed to Chinese state-sponsored groups. Our methodology integrates normal-log baselines, explicit validation cycles, and per-scenario coverage metrics, which are not documented in comparable datasets. These design choices strengthen reproducibility, reliability, and transparency, setting the Windows-APT Dataset 2025 apart from prior APT datasets.

In comparison to available datasets such as Linux-APT [2], CICIDS [3,4] and UNSW-NB15 [5], the Windows-APT Dataset 2025 offers broader scenario coverage, more comprehensive telemetry from both operating system and network layers, and rigorous validation against 36 APT-inspired scenarios. This combination provides researchers with realistic, high-fidelity data for testing, benchmarking, and developing advanced detection capabilities in Windows-based environments. Key benefits include:

• **Comprehensive scenario coverage:** Facilitates in-depth analysis of intrusion patterns and log sources specific to Windows, including a wide range of MITRE ATT&CK tactics and techniques.
• **Diverse research use cases:** Facilitates the creation of intrusion detection systems, compromise assessments, digital forensics investigations, and custom use cases to improve detection efficacy.
• **Rich multi-source telemetry:** Offers comprehensive simulated intrusions, malware execution traces, and payload activity captured through Sysmon and Wazuh, facilitating deep behavioral analysis.
• **Machine learning readiness:** Includes both benign and malicious traffic, enabling the training and testing of AI/ML models that can differentiate normal activity from intrusion attempts.
• **Structured mapping for reproducibility:** All events are systematically mapped to the MITRE ATT&CK framework, ensuring transparency and consistency across research applications.

By combining realistic APT emulation with extensive, well-structured logging, this dataset equips the research community with a robust foundation for studying complex adversary activities and enhancing defensive capabilities against evolving threats.

**Attribution note.** Scenario selection mirrors MITRE ATT&CK group entries *reported as China-attributed*; we do **not** assert attribution and focus strictly on reproducing reported TTPs for research.

In addition to general use cases, the supplementary files enable concrete, scenario-level analysis workflows. For example, researchers can use scenario_manifest.csv to filter logs by specific MITRE ATT&CK tactics or techniques, select scenarios associated with particular threat groups, and construct targeted evaluation subsets for intrusion detection research. Similarly, validation_summary.csv supports the assessment of scenario execution consistency and reproducibility by comparing expected versus observed technique execution rates across repeated runs. Together with the labeled ATT&CK mappings and scenario structure, these resources support practical workflows such as supervised and semi-supervised model training and ATT&CK-mapped anomaly detection pipelines.

## 2 Background

APTs are complex cyberattacks that involve long-term, planned campaigns aimed at breaching and compromising organizational networks [6]. Initially, they targeted military sites [7], but APTs have now spread to industries and governments worldwide [8,9]. Their multi-stage, persistent, and targeted nature makes them hard to detect and address [10]. Effective detection requires understanding the attack lifecycle, which is often represented using the MITRE ATT&CK® framework. This framework lists the techniques, tactics, and procedures used by adversaries in real-world attacks [11].

One ongoing issue in this area is the lack of detailed, Windows-specific benchmark datasets that cover the entire APT lifecycle. Existing datasets, including DARPA1998 [12], UNSW-NB15 [5], CIC-IDS2017[3] and CSE-CIC-IDS2018 [4], focus on network traffic but offer limited coverage at the host level. Newer datasets, like the Linux-APT Dataset [2], imitate APT behavior but fail to capture the wide range of techniques throughout the complete lifecycle in Windows settings. Reviews of APT datasets [13,14] highlight issues related to realism and completeness.

This led to the creation of a dataset focused on Windows, which aims to fill these gaps. It reproduces realistic APT scenarios in a controlled environment, generates multi-source telemetry, and systematically maps all activities to the MITRE ATT&CK framework.

## 3 Data Description

### 3.1 Instructions

The Windows-APT Dataset 2025 [1] has three main features. First, it contains scenarios inspired by 36 APT that come from MITRE ATT&CK® threat actor profiles, all recreated in a controlled Windows 10 environment. Second, it offers multi-source telemetry by combining host and network logs gathered through Wazuh and Sysmon. This approach captures attacker behavior in detail. Third, all activities are systematically mapped to the MITRE ATT&CK framework. This mapping ensures transparency and reproducibility, while also allowing for various research applications.

**Fig. 1:**
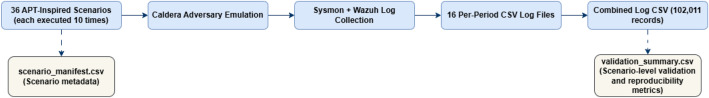
Simplified overview of the Windows-APT Dataset 2025 file structure.

The Windows-APT Dataset 2025 is provided in CSV format, making it readily usable for researchers developing and evaluating APT detection methods, including machine learning approaches. In total, the dataset contains approximately 102,000 log records, collected from three Windows agents monitored by Wazuh [15]. These logs were generated from 36 APT-inspired scenarios, which were derived from threat actor profiles documented in the MITRE ATT&CK database [11]. In designing the dataset, our primary focus was to reproduce their reported tactics, techniques, and procedures (TTPs) in a way that ensures both research transparency and reproducibility. Fig. 1 provides a simplified visual overview of the dataset file structure, illustrating how the simulated scenarios, log files, and supplementary metadata files are related.

To maximize coverage, the simulated scenarios cover several ATT&CK tactics, including Initial Access, Execution, Persistence, Privilege Escalation, Defense Evasion, Credential Access, Discovery, Lateral Movement, Command and Control, and Exfiltration. Every scenario is aligned with one or several ATT&CK techniques, which are detailed in a supplementary manifest file to help researchers quickly identify and analyze relevant behaviors.

Because of Wazuh’s extraction format, the dataset is organized into multiple CSV files. A consolidated file containing all log records is also included to support ease of access and broader analysis. This dual structure preserves detailed mappings between scenarios and MITRE ATT&CK TTPs while allowing researchers to perform both targeted and large-scale evaluations. In total, the dataset comprises 19 files, covering the period from October 28, 2024, to December 22, 2024, as summarized in Table 1.

**Table 1:** Summary of dataset files, including collection date ranges, number of log records, and file sizes.

S#	File Name (CSV)	Number of Records	File Size (KB)
1	01–03-December	8839	20,384
2	03–04-December	7292	17,175
3	04–07-December	7751	16,724
4	07–10-December	9186	20,199
5	10-December-P1	9050	19,312
6	10- December-P2	1189	2698
7	11–12-December	9656	22,167
8	12–13-December	6407	14,924
9	13–14-December	9739	21,492
10	14–17-December	5645	11,737
11	22-December	1174	2572
12	28-October-01-November	694	1396
13	01–11-November	8808	21,697
14	11–16-November	6555	10,535
15	23–30-November	8039	20,391
16	30-November	2003	4026
17	Combine	102,011	242,124
18	scenario_manifest.csv	37	28
19	validation_summary.csv	37	8

*Note:*Table 1 lists all 19 CSV files. Rows 1–17 are log CSVs (16 per-period plus one combined); rows 18–19 are supplementary CSVs (scenario_manifest.csv and validation_summary.csv).

Beyond the primary log data, the dataset includes two supplementary resources designed to strengthen reproducibility and enable scenario-level analysis:

• scenario_manifest.csv: Contains metadata for each of the 36 scenarios, including the scenario ID, name, associated MITRE group, reported origin, simulated tactics and techniques, expected artifacts (e.g., processes, network connections, file paths), and reference links. This allows researchers to cross-reference scenarios with threat intelligence sources and identify relevant behavioral patterns.
• validation_summary.csv: Provides a record of the validation process for each scenario, documenting the number of runs, expected technique count, average number of successfully executed techniques, success ratios, anomaly notes, secondary review outcomes, and validation check summaries. This file offers an understanding of the consistency and quality of the simulations, in addition to being documentation for scenario repeatability.

Collectively, these additional files represent a complete guide to understanding the dataset, making its construction transparent, and facilitating accurate replication of experiments.

All data, supplementary metadata files, and usage materials, including a README walkthrough and example Python scripts, are publicly available via the Mendeley Data repository [1].

### 3.2 Data format

Raw format: Comma-Separated Values (CSV)

### 3.3 Type of data

The dataset encompasses all essential fields required for a comprehensive analysis of APT behaviors. Key fields include Timestamp, Agent-Name, Full-log (detailing commands, payloads, and arguments), Rule Description, MITRE Tactic and Associated IDs, MITRE Technique, File Hash (MD5 and SHA256), Source-OS, Path, Source Datatype, Filename, Source-log, Rule PCI-DSS, Rule HIPAA, Rule NIST800–53, and Source/Destination IP & Port. These fields are critical for understanding the context and specifics of each simulated APT attack. A detailed summary of the dataset fields and their descriptions is presented in Table 2.

**Table 2:** Description of log entry fields contained in the Windows-APT Dataset 2025.

Field Name	Description
Timestamp	The date and time when the log entry was recorded.
Agent-Name	The name of the agent or system involved in the attack simulation.
Full-log	Complete log entry, including commands executed, payloads used, and arguments.
Rule Description	Description of the rule that triggered the log entry.
MITRE Tactic and Associated IDs	Tactics used in the attack as per the MITRE framework along with their IDs.
MITRE Technique	Specific techniques employed during the attack as per the MITRE framework.
File Hash (MD5)	MD5 hash of files involved in the attack for integrity verification.
File Hash (SHA256)	SHA256 hash of files involved in the attack for integrity verification.
Source-OS	Operating system from which the logs were generated.
Path	File path related to the log entry.
Source Datatype	Type of data source generating the log (e.g., application, system).
Filename	Name of the file associated with the log entry.
Source log	Original source log from which this entry was derived.
Rule PCI-DSS	Indicates compliance with PCI-DSS standards relevant to this log entry.
Rule HIPAA	Indicates compliance with HIPAA standards relevant to this log entry.
Rule NIST800–53	Indicates compliance with NIST 800–53 standards relevant to this log entry.
Source/Destination IP & Port	IP address and port information for both source and destination involved in the log entry.

Table 3 presents a representative example of a single log entry in the Windows-APT Dataset 2025. The Full-log field contains the raw Sysmon event as collected by Wazuh, while the MITRE Technique field indicates the ATT&CK technique associated with the observed behavior. File hash fields enable artifact correlation across processes and scenarios, supporting forensic analysis and reproducibility.

**Table 3:** Example Log Entry (Annotated).

Field	Example Value	Explanation
Timestamp	2024–12–03 20:30:50 UTC	Time when the event was recorded
Agent-Name	window-agent3	Windows host generating the event
EventID	11	Sysmon FileCreate event
Image	C:\Program Files\rempl\sedlauncher.exe	Process responsible for the action
TargetFilename	C:\Windows\Temp…\AppxProvider.dll	File created during execution
MITRE Technique	T1574.010	Services File Permissions Weakness
Full-log	File created: Image=sedlauncher.exe	Raw Sysmon event content
File Hash (SHA256)	e3b0c44298fc1c14…	Hash of the created file

## 4 Experimental Design, Materials and Methods

### 4.1 Environmental setup

**Fig. 2:**
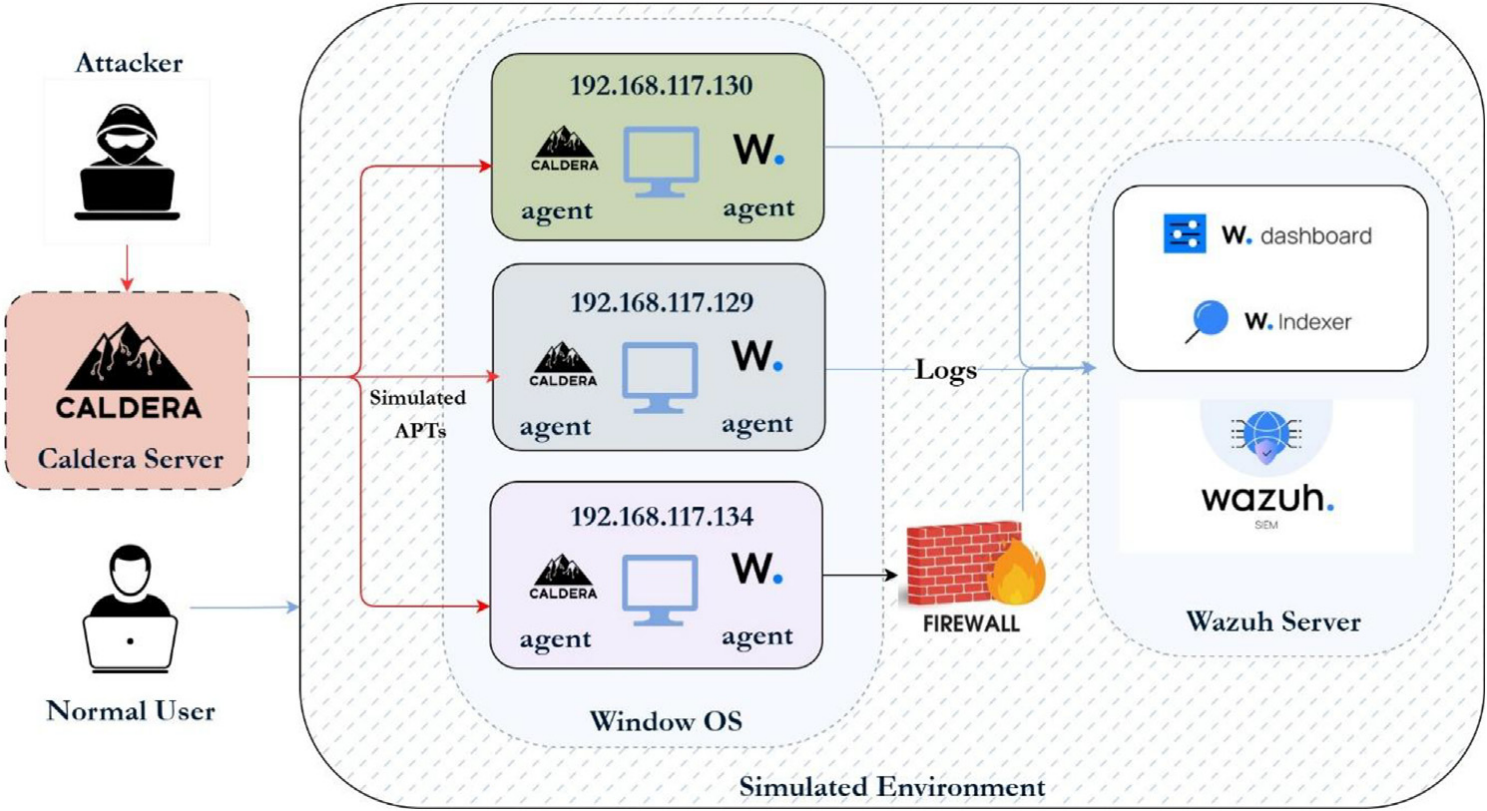
Environment architecture for Windows-APT. Caldera 5.0.0 orchestrates APT emulation on three Windows 10 hosts.

The infrastructure for the APT simulation is shown in Fig. 2. We chose Windows 10 as the main simulation platform because it is the operating system most often targeted in enterprise settings. It is also the main focus of APT tools and campaigns. Kaspersky notes, “Windows is, due to its popularity, the platform for which we discover most APT attack tools” [16]. In contrast, Linux is often seen as more secure and has dealt with fewer large-scale malware attacks. By focusing on Windows, our dataset becomes more relevant for defenders. Real-world APT actors regularly target Windows-based systems for persistence, credential theft, and lateral movement. Simulating scenarios in a Windows environment thus provides valuable research opportunities and matches the tactics used by actual adversaries.

The environment comprises three Windows systems specifically configured to simulate APTs. Logs are collected through Windows Event Logs in conjunction with Sysmon [17], which has been customized based on the MITRE framework to enhance visibility into system activities. Tailored Sysmon configurations [18] enable the detection of tactics and techniques outlined in the MITRE ATT&CK framework. Wazuh serves as the centralized data collection platform, functioning as a SIEM system. This setup captures both network-based and operating-system-based logs, forwarding them to the Wazuh server for comprehensive analysis. For adversary emulation, the Caldera framework [19] developed by MITRE is employed, providing tools to effectively simulate the TTPs used by real-world APTs. The primary objective of this configuration is to systematically observe and document the behavior and tactics employed by APTs within a controlled environment. By capturing detailed logs and leveraging Caldera for simulation, the setup facilitates the generation of insights that inform future research directions and enhance existing security measures against APTs. To support reproducibility, the minimal configuration resources used during data generation are publicly available. Specifically, the Sysmon configuration file [18] and Wazuh rule sets [20] employed for host-level telemetry collection are provided through a public GitHub repository referenced in the manuscript. The Caldera adversary profiles used to execute each scenario are described at a high level to clarify scenario execution logic and expected observable behaviors, without requiring access to the full operational templates. In addition, a concise step-by-step reproduction checklist is included to summarize the experimental workflow, including environment setup, agent deployment, scenario execution, and log extraction. The reproduction workflow consists of the following high-level steps:

• Preparation of a Windows 10 virtual machine and baseline snapshot.
• Deployment and configuration of Caldera, Sysmon, and Wazuh agents.
• Execution of the selected APT-inspired scenarios with repeated runs.

Collection, aggregation, and export of logs into CSV files.During the data collection phase, several key considerations were prioritized:

• The primary goal was to capture a comprehensive set of logs from the Windows system, including network logs, OS logs, file and process activities, images, and services. This was accomplished by simulating APT attacks based on the MITRE ATT&CK framework.
• APTs are multi-step by nature. Therefore, the dataset includes logs tagged with relevant tactics and techniques for each phase of the attack lifecycle. This involved simulating a variety of APTs and capturing logs with timestamps to facilitate detailed analysis of their behaviors and applicability in machine learning models. The dataset is approximately 102,000 records which includes both benign and malicious logs.
• We emulate all tactics and techniques documented for each selected group where Caldera provides a reasonable ability. Logged coverage reflects what our pinned Sysmon configuration and Wazuh mappings actually captured on Windows hosts. As a result, some tactics that were simulated do not appear in the final telemetry. In this release, nine enterprise tactics are observed in logs; scenario-level intent remains documented in scenario_manifest.csv for transparency.

### 4.2 Configuration of Wazuh

The Wazuh architecture consists of three key components: the Dashboard, Indexer, and Server. The Wazuh Server serves as the central management unit, responsible for storing configurations and security rules essential for effective monitoring and analysis. It processes data received from agents deployed across endpoints, ensuring comprehensive coverage of security events. The Indexer plays a critical role in aggregating security data, such as alerts and system events, enabling efficient organization and retrieval. The Dashboard provides an intuitive interface for security analysts to visualize and interpret collected data, supporting real-time monitoring, report generation, and detailed threat analysis [15]. Together, these components form a robust SIEM solution that significantly enhances an organization's ability to detect and respond to APTs and other security incidents. Furthermore, Wazuh's integration with the MITRE ATT&CK framework enables tailored detection rules, improving the contextual understanding of attack patterns and strengthening overall cybersecurity defenses.

For Windows systems, the Wazuh configuration includes the installation of Wazuh agents on eachmachine, enabling comprehensive monitoring and logging of system activities. Logs collected by the agents are transmitted to a centralized Wazuh server, functioning as a SIEM system, to facilitate real-time analysis and correlation of events across multiple endpoints. By employing specific Wazuh rules [20], the system is configured to detect tactics and techniques defined in the MITRE ATT&CK framework, enabling the identification of potential APT behaviors through detailed event and log data. This integration enhances the detection of suspicious activities, improves incident response capabilities, and provides valuable insights for forensic analysis. This robust configuration ensures that all relevant logs are effectively captured, analyzed, and stored, thereby contributing to a stronger security posture against APTs targeting Windows environments. Our methodology also integrates normal-log baselines, explicit validation cycles, and per-scenario coverage metrics, which are not documented in comparable datasets.

### 4.3 Activity date range

All logs were collected between October 28, 2024 and December 22, 2024. Each scenario was executed 10 times on fresh Windows 10 snapshots across three Wazuh agents. Caldera 5.0.0 orchestrated runs, and Wazuh 4.5.2 ingested host and network telemetry. Pinned configurations and manifests are included in the repository for exact replication.

### 4.4 Key steps and tools used

The dataset is organized to include logs generated by Wazuh version 4.5.2 after simulating 36 APT-inspired attack scenarios based on techniques and tactics outlined in the MITRE ATT&CK framework [11]. Scenario selection began with a systematic review of all threat actor profiles in the MITRE ATT&CK database, filtering for those explicitly listed by MITRE as associated with Chinese state-sponsored groups. This list was cross-referenced with open-source threat intelligence reports from FireEye/Mandiant, Recorded Future, and other vendor analyses to corroborate attribution.

Groups that lacked enough publicly available technical details to reproduce their documented ATT&CK techniques were excluded. This decision ensured scenario fidelity and reproducibility. The dataset reflects the associations reported by MITRE, but it does not claim any specific nation-state attribution. Geopolitical attribution is naturally uncertain and may change as new information arises. For each simulated scenario, the metadata includes the MITRE Group ID, reported geographic origin, simulated tactics, associated ATT&CK techniques, and expected artifacts, as documented in scenario_manifest.csv. This format allows researchers to link simulated activity directly to documented adversary behaviors while keeping attribution sources transparent. *An overview of the simulated threat groups, their associated MITRE ATT&CK tactics and techniques, and high-level behavioral descriptions is provided in* Table 4*.*

**Table 4:** MITRE-listed China-attributed threat actor profiles (as publicly reported) and descriptions. attribution is *not* asserted by the authors. Information adapted from the MITRE ATT&CK Groups database [11] and open-source threat intelligence reports.

APT Group	Alternative Names	Description
APT1	-	APT1 is known for its cyber espionage activities targeting various industries, particularly in the U.S.
APT3	-	APT3 is associated with advanced cyber operations, often targeting technology and defense sectors.
APT5	-	APT5 focuses on espionage against telecommunications and technology companies, primarily in the U.S.
APT12	-	This group is known for its cyber espionage efforts against organizations in multiple sectors.
APT19	-	Known for targeting intellectual property and sensitive data from various sectors, including technology.
APT30	-	This group conducts espionage activities against government and private sector entities in Asia.
APT41	-	Involved in both cyber espionage and financially motivated attacks, targeting various sectors globally.
admin@338	-	Known for its sophisticated attacks against various industries, leveraging advanced malware techniques.
Aquatic Panda	-	Targets organizations in the telecommunications sector, focusing on espionage and data theft.
Axiom	-	Engages in cyber espionage primarily against entities in the technology sector.
BlackTech	-	Known for targeting technology companies and government agencies, often using APTs.
Chimera	-	Engages in cyber espionage with a focus on political and military targets within Asia.
Cinnamon Tempest	-	Targets organizations in the telecommunications sector, often using sophisticated phishing techniques.
Deep Panda	-	Known for its focus on cyber espionage against governmental organizations and private enterprises.
Elderwood	-	Engages in targeted attacks against high-profile entities, often using zero-day vulnerabilities.
GALLIUM	-	Focuses on espionage activities against telecommunications and technology companies worldwide.
IndigoZebra	-	Known for targeting organizations involved in high-tech industries, particularly in Asia-Pacific regions.
LuminousMoth	-	Targets a variety of sectors including defense and technology, employing sophisticated malware strategies.
menuPass	-	Engages in attacks primarily focused on stealing sensitive information from targeted organizations.
Mofang	-	Known for its cyber espionage activities targeting government and military sectors across Asia.
Naikon	-	Focuses on intelligence gathering from government entities, particularly in Southeast Asia.
PittyTiger	-	Engages in targeted attacks against educational institutions and research organizations.
Earth Lusca	TAG-22, CHROMIUM, ControlX, Charcoal Typhoon	Engaged in a range of cyber operations, including espionage against governmental entities worldwide.
Suckfly	-	Known for its focus on financial gain through cyber attacks against various sectors.
TA459	-	Engages primarily in espionage activities targeting governmental organizations in Asia-Pacific regions.
Winnti Group	-	Focuses on cyber operations aimed at stealing intellectual property from gaming and software companies.
ZIRCONIUM	APT31, Violet Typhoon	Engaged in espionage activities targeting governmental and private sector entities across Asia-Pacific regions.
Mustang Panda	A416, BRONZE PRESIDENT, RedDelta	Known for its cyber operations focusing on political targets within Asia, often employing advanced techniques.
BRONZE BUTLER	REDBALDKNIGHT, Tick	Targets critical infrastructure sectors with a focus on intelligence gathering through sophisticated methods.
Leviathan	-	Focuses on maritime-related targets as well as government agencies involved in international affairs.
Ke3chang	APT15, Mirage, Vixen Panda, GREF, Playful Dragon, Nylon Typhoon, RoyalAPT, NICKEL	Known for its extensive targeting of governmental organizations across multiple regions for intelligence purposes.
Rocke	-	Primarily focused on cryptocurrency-related attacks to gain financial advantage through illicit means.
Putter Panda	-	Engaged primarily in espionage activities targeting academic institutions and research facilities.
Volt Typhoon	BRONZE SILHOUETTE	Focuses on intelligence gathering from critical infrastructure sectors through sophisticated attack methods.
Tonto Team	-	Engages in targeted attacks against various sectors with a focus on data exfiltration.
Threat Group-3390	Earth Smilodon, TG-3390, Emissary Panda, APT27, Iron Tiger, LuckyMouse, BRONZE UNION	Involved in a variety of cyber operations, including espionage and data theft from high-profile targets globally.

In the MITRE ATT&CK framework, ``groups'' refer to clusters of activity tracked by a common name within the security community. Analysts may monitor these clusters using different methods, leading to similar activities being labeled differently across organizations. This can result in various aliases for the same or overlapping threat activities. To mimic attacker behavior, we used the Caldera framework version 5.0.0. It incorporates the specific tactics and techniques of each APT profile. All phases of each attack were simulated on fresh Windows 10 virtual machines, which represent a modern enterprise environment, using the Caldera agent. Wazuh was set up with custom detection rules to capture the complete range of simulated activity, enabling real-time alerting and detailed forensic analysis. This integrated approach improves the dataset’s value for threat detection research and operational testing.

To show the MITRE ATT&CK coverage of these simulations, Table 5 presents an excerpt from the dataset’s scenario_manifest.csv. It displays mappings between threat actors, tactics, techniques, and expected artifacts. The complete mapping for all 36 scenarios is available in the dataset repository.

**Table 5:** Excerpt from scenario_manifest.csv showing scenario-to-technique coverage for selected APT simulations.

Scenario Name	MITRE Group ID	Tactics (MITRE)	Techniques (MITRE ID)	Expected Artifacts
APT30	G0013	Execution; Initial Access	T1204.002; T1566.001	net: HTTPS 443; file: C:\Users\Public\art.jse
Elderwood	G0066	Command & Control; Defense Evasion	T1027.002; T1105; T1204.002	net: HTTP 80; file: %TEMP %\OSTapGet.js
APT41	G0096	Collection; Credential Access; Discovery	T1001.002; T1003.001; T1059.003	proc: powershell.exe; net: SMB 445
Mustang Panda	G0129	Collection; Credential Access; Execution	T1005; T1012; T1059.005	file: phishing_docs.docm; removable_media.exe

Coverage percentages came from dividing the number of unique MITRE ATT&CK techniques successfully used in each scenario by the total number of techniques linked to that scenario in MITRE’s database. This creates a clear and reproducible link between simulated actions and documented enemy behaviors.

**Note:** This table is an excerpt. The complete mapping of scenarios to techniques for all 36 APT simulations is included in the dataset’s scenario_manifest.csv file. TTP coverage percentages were calculated by dividing the number of successfully executed MITRE ATT&CK technique instances in each scenario by the total number of expected techniques for that scenario.

**Fig. 3:**
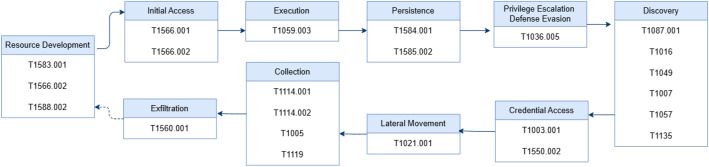
APT1 emulation flow. The diagram reflects simulated tactics and techniques based on ATT&CK profiles. Logged coverage may differ, depending on Caldera ability execution and host-side visibility of Sysmon and Wazuh.

As an example, Fig. 3 shows the attack flow for Scenario S03 (APT1). This scenario includes multiple tactics, such as Initial Access, Execution, Discovery, Credential Access, Defense Evasion, Lateral Movement, and Collection. The techniques used are listed under each tactic, and the artifacts produced are examples of those collected from all 36 scenarios in our dataset.

### 4.5 Validation and quality control

To ensure the reliability and scientific quality of the Windows-APT Dataset 2025, each of the 36 APT-inspired scenarios went through a thorough validation process before being included in the final dataset. We defined the anticipated tactics, techniques, and observable artifacts, such as process events, registry changes, and network flows, based on MITRE ATT&CK documentation and publicly available threat intelligence reports.

Each scenario was run 10 times against clean Windows snapshots to check for repeatability and reliability of outcomes. We measured stability using the success ratio, which indicates the percentage of expected techniques that successfully executed in each run. The success ratios for all scenarios varied from 75 % for Elderwood, Mofang, Putter Panda, and TA459 to 100 % for APT 30, APT12, IndigoZebra, and PittyTiger. The overall average success ratio was 83.58 %, with a standard deviation of (±7.02 %), indicating high reproducibility. Lower success ratios, like Elderwood’s 75 %, largely reflect the operational complexity of some multi-stage techniques and occasional timing or execution constraints specific to the Caldera framework. These findings reflect the challenges of certain multi-stage techniques and timing issues rather than missing or poorly simulated techniques. Such limitations are common in adversary emulation platforms and are documented here for transparency. On average, scenarios involved 24.89 expected techniques, and 18.58 were successfully executed per run. Scenarios with success ratios below 85 % were reviewed manually, with deviations linked to minor timing issues with the Caldera agent or environmental differences, rather than the absence of intended techniques.

We manually checked the Caldera operation status and Wazuh logs after each run to confirm the triggering of intended TTPs and the presence of expected system and network artifacts. Some benign system activities, like legitimate command-line executions or registry changes, also generated MITRE-tagged events. We kept these to maintain operational realism, but machine learning researchers should take them into account when addressing potential false positives.

The companion file, validation_summary.csv, includes complete per-scenario results. This file details the number of runs, expected techniques, average techniques executed, success ratios, anomalies, and validation checks. This transparency supports reproducibility and allows for detailed comparisons in future research. All experiments can be replicated on a fresh Windows 10 virtual machine using the provided configuration scripts along with the documented Caldera and Wazuh settings.

### 4.6 General vs malicious logs

The dataset includes both malicious and normal logs collected by Wazuh. Malicious logs represent activities associated with APT scenarios and are tagged with MITRE ATT&CK tactic and technique identifiers. For example, during the APT41 simulation, logs indicating credential dumping from LSASS were tagged with T1003.001 under the Credential Access tactic. In contrast, normal logs, which may also contain MITRE tags, capture legitimate user activities or potentially suspicious but benign actions.

**Fig. 4:**
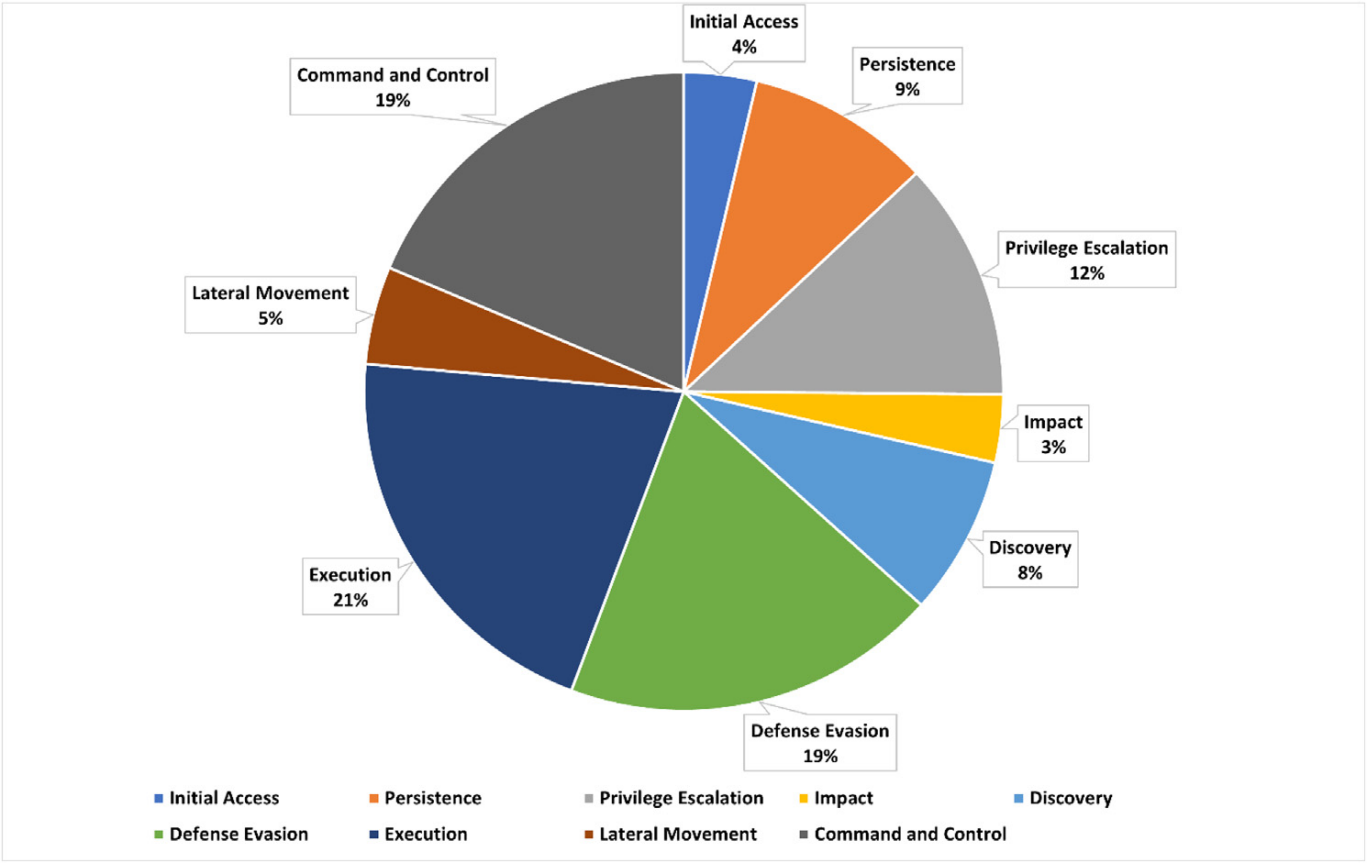
Distribution of observed ATT&CK Enterprise tactics in logged telemetry (nine tactics).

Both log types are essential for developing machine learning models and anomaly detection methods because they provide a balanced representation of true positives and realistic false positives. The dataset reports per-tactic and per-technique counts for the logged telemetry, as illustrated in Fig. 4 and summarized in Table 6, Table 7. In total, 1024 technique instances were executed across all scenarios. The logged telemetry covers nine enterprise tactics with the following distribution: Execution (21 %), Defense Evasion (19 %), Command and Control (19 %), Discovery (17 %), Persistence (9 %), Lateral Movement (7 %), Privilege Escalation (5 %), Initial Access (2 %), and Impact (1 %). We attempted to emulate all tactics reported for the selected groups; under the pinned Sysmon and Wazuh configurations, nine tactics produced MITRE-mapped events in logs. Credential Access and Exfiltration were simulated where applicable but did not yield observable events in the final telemetry.

**Table 6:** Summary of techniques and their counts.

Technique ID	Description	Count
T1105	Ingress Tool Transfer	18,308
T1059.003	Windows Command Shell	12,174
T1112	Modify Registry	8867
T1087	Account Discovery	7944
T1570	Lateral Tool Transfer	4484
T1078	Valid Accounts	3626
T1053.005	Scheduled Task	3031
T1059	Command and Scripting Interpreter	3001
T1055	Process Injection	2517
T1565.001	Stored Data Manipulation	2512
T1070.004	File Deletion	2082
T1059.001	PowerShell	1936
T1574.001	DLL Search Order Hijacking	1422
T1543.003	Windows Service	899
T1485	Data Destruction	775
T1027	Obfuscated Files or Information	612
T1027.004	Compile After Delivery	612
T1021.006	Windows Remote Management	372
T1574.002	DLL Side-Loading	305
T1548.002	Bypass User Account Control	227
T1059.005	Visual Basic	193
T1070	Indicator Removal on Host	80
T1546.011	Application Shimming	79
T1548	Abuse Elevation Control Mechanism	74
T1531	Account Access Removal	72
T1562.001	Disable or Modify Tools	59
T1095	Non-Application Layer Protocol	57
T1574.010	Services File Permissions Weakness	46
T1562.004	Disable or Modify System Firewall	43
T1098	Account Manipulation	40
T1110	Brute Force	34
T1489	Service Stop	31
T1210	Exploitation of Remote Services	28
T1547.001	Registry Run Keys / Startup Folder	11
T1003.002	Security Account Manager	10
T1546.003	Windows Management Instrumentation Event Subscription	8
T1047	Windows Management Instrumentation	7
T1529	System Shutdown/Reboot	7
T1484	Domain Policy Modification	6
T1036	Masquerading	6
T1110.001	Password Guessing	3
T1003.001	LSASS Memory	2
T1546	Event Triggered Execution	2
T1218	System Binary Proxy Execution	2
T1136	Create Account	1

*Note:* Counts in Table 6 follow sub-technique granularity when applicable. Parent techniques and sub-techniques are counted separately unless stated otherwise.

**Table 7:** Counts of MITRE ATT&CK tactics represented in logged telemetry (nine enterprise tactics).

MITRE Tactic	Count
Initial Access	3626
Persistence	9165
Privilege Escalation	11,948
Impact	3397
Discovery	7944
Defense Evasion	18,776
Execution	20,342
Lateral Movement	4884
Command and Control	18,365

This categorization improves understanding of APT behaviors and highlights class imbalance that practitioners should address via resampling, data augmentation, or class weighting when training detection models. Table 8 further summarizes the dataset by detailing counts of malicious and normal logs collected during the simulations. All experiments can be reproduced on a fresh Windows 10 virtual machine using the provided configuration scripts and the documented Caldera and Wazuh settings. Counts in Table 6 follow sub-technique granularity when applicable, and per-scenario intent, expected tactics and techniques, and observed coverage are provided in the supplementary file scenario_manifest.csv for full transparency.

**Table 8:** Distribution of general versus malicious logs captured in the Windows-APT Dataset 2025.

	General	Malicious
Count	38,393	63,620

Across all scenarios, the dataset covers 45 MITRE ATT&CK techniques spanning nine enterprise tactics. Coverage was computed as the proportion of techniques documented by MITRE for each group that we successfully emulated (or substituted with Caldera-equivalent abilities when a direct module was unavailable) and captured in Wazuh logs. Per-scenario coverage appears in scenario_manifest.csv.

### 4.7 Comparison

Table 9 compares our dataset with existing datasets focused on APT-related activity. Most prior datasets focus on capturing network traffic, with notable exceptions like the LANL dataset [13] from 2018 and the Linux-APT dataset [1] published in 2024. The Linux-APT dataset only covers four simulated APT attacks, which significantly limits its scope and generalizability. In contrast, our dataset uniquely simulates 36 APT-inspired scenarios. These scenarios are systematically derived from MITRE ATT&CK threat actor profiles. Unlike Linux-APT, which is limited to a Linux environment, Windows-APT targets the Windows operating system, a platform often targeted in real-world APT campaigns. This broader scope, along with our methodology that integrates normal-log baselines, validation cycles, and per-scenario coverage metrics, allows for a deeper and more reproducible analysis of APT behaviors. By addressing these gaps, the Windows-APT Dataset 2025 creates a solid foundation for future research and better detection methods in Windows environments.

**Table 9:** Comparison of Different IDS Datasets for APT.

S#	Dataset	Year	Dataset Type	Duration	Windows OS support	References
1	Linux-APT	2024	Network and OS Logs	98-Days	No	[2]
2	CSE-CIC-IDS2018	2018	Network and OS Logs	15-Days	Yes	[4]
3	CIC-IDS2017	2017	Network-based Intrusions	5-Days	Yes	[3]
4	UNSW-NB15	2015	Network-based Intrusions	31-Hours	Yes (limited)	[5]
5	LANL Dataset	2018	Network and OS Logs	90-Days	Yes	[13]
6	TRAbID	2017	Network-based Intrusions	8-Hours	Yes	[14]

### 4.8 Differentiation from existing datasets

While our dataset and the Linux-APT dataset [2] have a similar high-level structure and share common adversary emulation tools like Caldera and Wazuh for data collection, there are several key differences that make the Windows-APT Dataset 2025 unique and complementary:

• Operating system focus: The Linux-APT dataset only targets Linux environments, while our dataset is specifically designed for Windows 10 systems. This platform is more often targeted by APT campaigns, according to various threat intelligence reports.
• Scenario coverage: Linux-APT includes only 4 APT-inspired attack scenarios. In contrast, Windows-APT simulates 36 scenarios based on all the known China-attributed profiles documented in MITRE ATT&CK as of 2024. This ensures broader coverage of adversary behaviors.
• TTP diversity: Our logged telemetry spans nine ATT&CK Enterprise tactics and 45 distinct techniques and sub-techniques (Table 6), enabling a detailed view of attacker behaviors on Windows hosts.
• Validation rigor: Each scenario in our dataset was executed 10 times. We provide validation results in a supplementary file (validation_summary.csv) to ensure reproducibility and data reliability. This level of quality control was not documented in Linux-APT.
• Dataset size and richness: Windows-APT contains about 102,000 log records across 17 log CSVs (plus two supplementary CSVs), combining benign and malicious telemetry for realistic training and evaluation of detection models.

These differences show that the Windows-APT Dataset 2025 is not just a repeat of past work; it is an extension that addresses a critical gap in publicly available datasets for Windows-targeted APT research. Our methodology also includes normal-log baselines, explicit validation cycles, and per-scenario MITRE ATT&CK coverage metrics. These elements are not documented in similar datasets like Linux-APT or related emulation collections.

## Limitations

The Windows-APT Dataset 2025 focuses only on Windows environments. This allows for realistic modeling of APT behavior in systems that are commonly targeted. However, it does not capture tactics on other platforms like Linux, macOS, or mobile operating systems.

The dataset shows the natural frequency of tactics observed in real campaigns, leading to class imbalance. Common stages like Discovery, Defense Evasion, and Execution appear more often than less common techniques like Masquerading or Application Shimming. Two enterprise tactics (Credential Access and Exfiltration) do not appear in the logged telemetry due to emulation or logging limits in the selected Caldera profiles and pinned Sysmon/Wazuh configurations. We plan to expand host visibility and rules to increase observed coverage in a future update. Researchers applying supervised learning may need balancing or augmentation strategies.

Although the Windows-APT Dataset 2025 provides comprehensive host-level telemetry for a wide range of ATT&CK tactics and techniques on Windows systems, a key limitation relates to host visibility constraints inherent to Sysmon- and Wazuh-based logging. Advanced attack techniques relying on in-memory execution, kernel-level exploitation, or firmware-level persistence are either partially observable or entirely invisible at the host logging level. Such behaviors typically leave minimal or no artifacts in standard event logs, and volatile memory is not captured by default in the logging pipeline. In addition, the Caldera adversary emulation framework does not fully support certain low-level or stealthy techniques, which further constrains observable coverage. In some cases, functionally equivalent modules were used to approximate techniques that were not directly available.

Finally, while scenarios were recreated under controlled conditions to enhance reproducibility, real-world APT activity may differ due to variations in infrastructure and the adaptive behavior of adversaries. An additional limitation relates to the scope of scenario selection. The simulated attack scenarios were deliberately inspired by publicly documented MITRE ATT&CK group reports commonly attributed to China in open-source intelligence, with the goal of reproducing well-documented tactics and techniques in a controlled and reproducible setting rather than asserting real-world attribution. This scoped focus does not represent the full diversity of global APT activity. Future extensions of the dataset may incorporate a broader range of threat groups, attack tools, environments, and operating system versions to further improve coverage and generalizability

## Ethics Statement

The authors confirm that this work complies with the ethical requirements for publication in *Data in Brief*. This study does not involve human subjects, animal experiments, or the collection of data from social media platforms.

## CRediT authorship contribution statement

**Maryam Mozaffari:** Methodology, Writing – original draft. **Abbas Yazdinejad:** Validation, Investigation, Writing – review & editing. **Ali Dehghantanha:** Supervision, Investigation.

## Data Availability

Mendeley DataWindows-APT 2025: A Dataset for APT-Inspired Attack Scenarios on Windows Systems (Original data). Mendeley DataWindows-APT 2025: A Dataset for APT-Inspired Attack Scenarios on Windows Systems (Original data).

## Appendix Supplementary materials

Image, application 1

Image, application 2
